# Glucosyl Platinum(II) Complexes Inhibit Aggregation of the C-Terminal Region of the Aβ Peptide

**DOI:** 10.1021/acs.inorgchem.1c03540

**Published:** 2022-02-16

**Authors:** Sara La Manna, Marilisa Leone, Ilaria Iacobucci, Alfonso Annuziata, Concetta Di Natale, Elena Lagreca, Anna Maria Malfitano, Francesco Ruffo, Antonello Merlino, Maria Monti, Daniela Marasco

**Affiliations:** † Department of Pharmacy, University of Naples “Federico II”, 80131 Naples, Italy; ‡ Institute of Biostructures and Bioimaging - CNR, 80134 Naples, Italy; § Department of Chemical Sciences, University of Naples “Federico II”, 80126 Naples, Italy; ∥ CEINGE Biotecnologie Avanzate S.c.a r.l., “University of Naples Federico II”, 80131 Naples, Italy; ⊥ Interdisciplinary Research Centre on Biomaterials (CRIB), Department of Ingegneria Chimica del Materiali e della Produzione Industriale (DICMAPI), University “Federico II”, 80125 Naples, Italy; # Department of Translational Medical Science, University of Naples “Federico II”, 80131 Naples, Italy

## Abstract

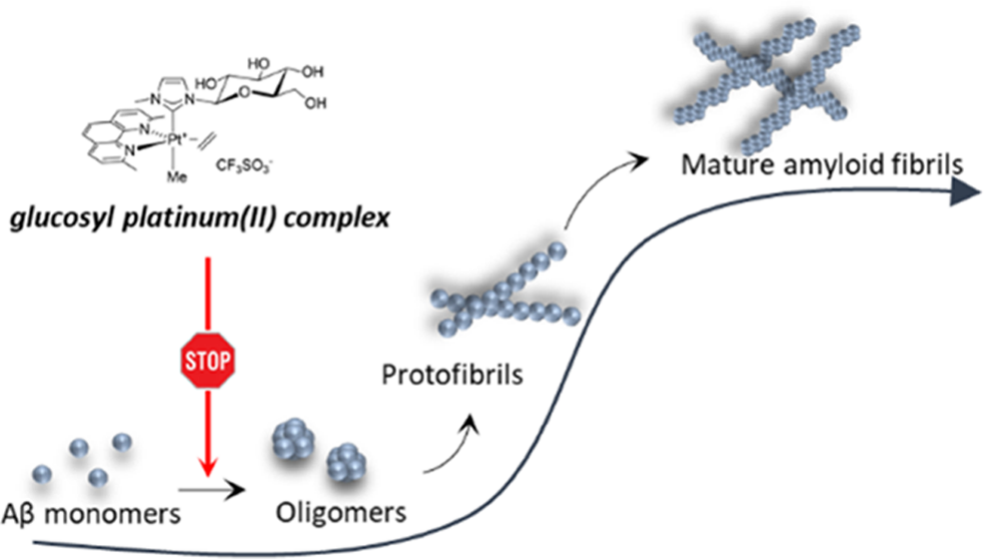

Neurodegenerative diseases are often caused by uncontrolled amyloid aggregation. Hence, many drug discovery processes are oriented to evaluate new compounds that are able to modulate self-recognition mechanisms. Herein, two related glycoconjugate pentacoordinate Pt(II) complexes were analyzed in their capacity to affect the self-aggregation processes of two amyloidogenic fragments, Aβ_21–40_ and Aβ_25–35_, of the C-terminal region of the β-amyloid (Aβ) peptide, the major component of Alzheimerʼs disease (AD) neuronal plaques. The most water-soluble complex, **1Pt**_**dep**_, is able to bind both fragments and to deeply influence the morphology of peptide aggregates. Thioflavin T (ThT) binding assays, electrospray ionization mass spectrometry (ESI-MS), and ultraviolet–visible (UV–vis) absorption spectroscopy indicated that **1Pt**_**dep**_ shows different kinetics and mechanisms of inhibition toward the two sequences and demonstrated that the peptide aggregation inhibition is associated with a direct coordinative bond of the compound metal center to the peptides. These data support the *in vitro* ability of pentacoordinate Pt(II) complexes to inhibit the formation of amyloid aggregates and pave the way for the application of this class of compounds as potential neurotherapeutics.

## Abstract 2

Two related glycoconjugate pentacoordinate Pt(II) complexes are able to modulate the self-aggregation mechanism of two amyloidogenic fragments, Aβ_21−40_ and Aβ_25−35_, of the β-amyloid peptide, the major component of neuronal plaques in Alzheimerʼs disease, corroborating the potential application of transition-metal complexes as therapeutics in neurodegenerative diseases.

## Introduction

In Alzheimerʼs disease (AD) pathophysiology, the β-amyloid (Aβ) peptide represents the prevalent component of senile plaques^1^ even if it is present at the early phases of life, and in its monomeric form, it can act as a positive regulator of the presynaptic release in hippocampal neurons.^2^ Aβ is a polypeptide spanning 1–40 or 42 residues that is mostly intrinsically disordered.^3^ Through a self-assembly process that is typical of amyloid aggregation, it forms different Aβ oligomers, endowed with diverse levels of order that represent key pathogenic species in AD.^4^ Amyloid assemblies induce synaptic dysfunction and neuronal death^5^ and oxidative damage and inflammation, which further corroborate the progression of the disease.^6^ The presence of metals like Zn, Fe, Cu, and Al inside amyloid plaques enhances Aβ-induced oxidative damage and its aggregation level; thus, a chelation approach to directly target metals in the brain can be conceived as a way to reduce harmful consequences of metal/fibril accumulation.^7^ However, the most powerful therapeutic approach for AD is based on the inhibition of the aggregation of the Aβ peptide^8^ and great efforts have been devoted to the identification of molecules capable of inhibiting its self-recognition. It has been shown that these compounds can have different origins (synthetic, natural) and chemical nature (phenols, peptide, antibodies, small molecules, *etc*.).^9−12^ Even though many trials are actually ongoing, especially on natural compounds,^13^ no drugs have entered into clinical use yet: this can be mainly due to the inability of targeting protein interfaces without regular secondary structures that could be assumed as templates to design inhibitors.^14,15^ Recently, multivalent systems (as dendrimers) were investigated to gain access to different protein subregions.^16^ Indeed, different Aβ regions contribute to amyloid aggregation: the N-terminus,^17^ hydrophobic core,^18^ so-called hinge and turn regions,^19^ and C-terminus.^20,21^ Experimental data indicated that the C-terminal region of Aβ can be addressed by the cyclohexanehexol scaffold: indeed, the scyllo-inositol compound interferes with the fibrillization process and competes with endogenous phosphatidylinositol for binding to the Aβ polypeptide, appearing as a promising therapeutic agent, currently in Phase II trials.^22^

The unique properties exhibited by transition-metal complexes as drugs, including their tunability in the oxidation and the coordination states, allow them to enter into many pharmaceutical applications.^23−25^ In the amyloid context, they can be considered as good starting compounds for the development of novel neuroprotective agents.^26,27^ The stability and inertness of Pt(II) and Ru(III)^28^ complexes allowed a wide range of investigations with different amyloid systems: phenanthroline (phen)–Pt(II) complexes with two monodentate ligands (*e.g.*, chlorides) inhibit the aggregation of Aβ_1–40_ and its N-terminal region^29−31^ as well as of prion protein (PrP) fragments.^32,33^ Polyoxometalate derivatives of Pt^34^ and V^35^ suppress amyloid aggregation in a mechanism involving multiple interactions: (i) the coordination of the metal, (ii) electrostatic, (iii) hydrogen, and (iv) van der Waals forces. Octahedral Co compounds,^36^ as well as square-planar Pt complexes bearing polyaromatic ligands, interact with the Aβ peptide *via* π–π stacking.^37^ Furthermore, hetero-multinuclear Pt–Ru complexes are able to revert amyloidosis: they regulate amyloid-induced cytotoxicity in insulinoma β-cells and significantly increase cell viability.^38^ Traditionally, due to the presence of His residues in its sequence, the N-terminal region of the Aβ peptide was considered the main target for inhibition studies of Aβ aggregation by metal compounds.^39^ Conversely, with the aim to deepen the druggability of C-terminus by transition-metal complexes, in our recent investigations we assumed as amyloid model the fragment spanning residues 21–40 of Aβ (Aβ_21–40_, Table 1),^40−42^ which is also deeply involved in the mechanism of aggregation of the entire Aβ peptide. Herein, we also studied the behavior of a shorter Aβ fragment spanning residues 25–35 (Aβ_25–35_, Table 1). It has been shown that this peptide is the most cytotoxic among the known Aβ peptides.^43^

**Table 1:**
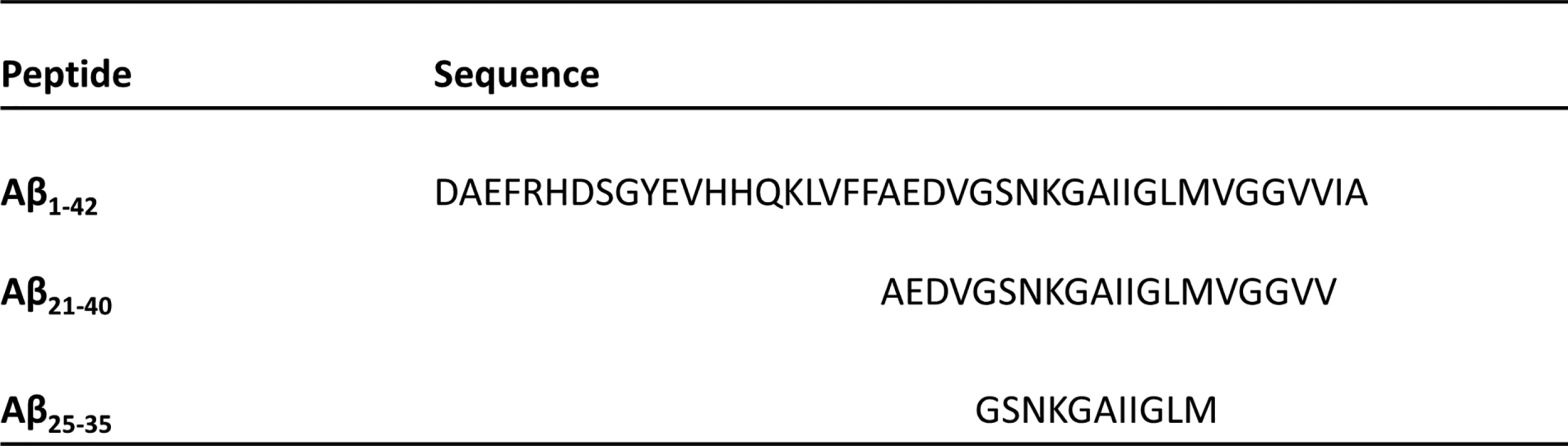
Sequences of the Aβ_1-42_ Peptide and Peptides Investigated in this Study and Derived from its C-Terminal Domain

**Figure 1:**
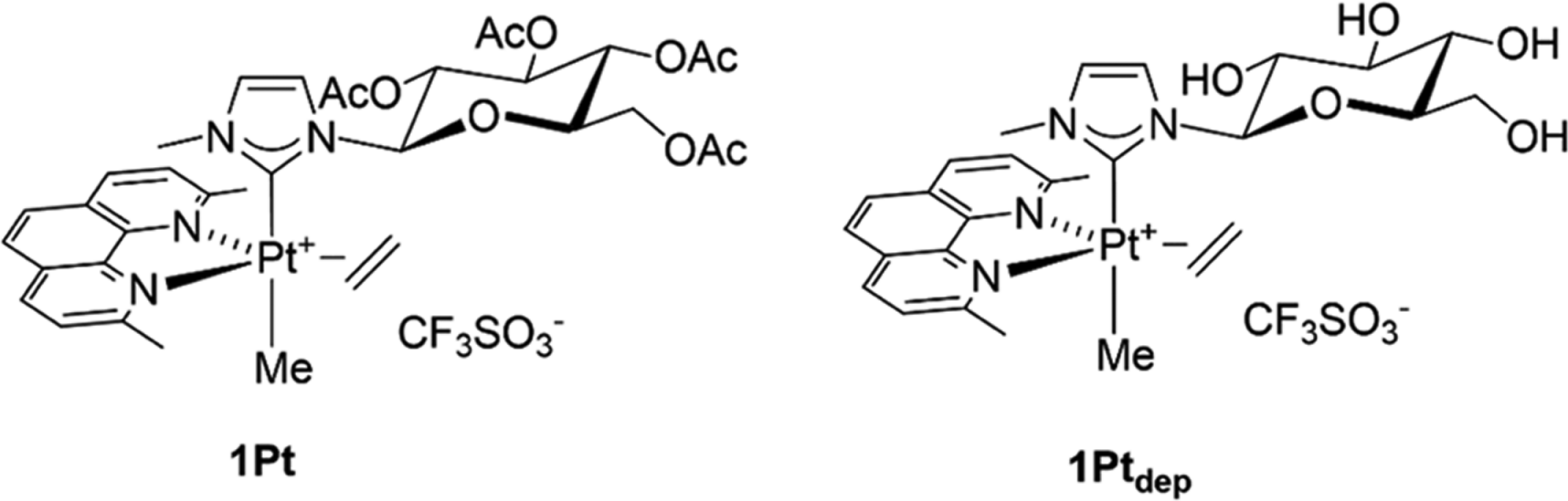
Pentacoordinate platinum(II) complexes **1Pt** and **1Pt**_**dep**_.

Very recently, we have investigated the ability of a series of square-planar Pt(II) complexes to inhibit the aggregation of amyloid peptides.^40−42^ Among the investigated systems, the pyridine-based platinum(II) complex, called Pt-terpy, exhibited good inhibitory effects of amyloid aggregation, also allowing, for its better water solubility when compared to other investigated complexes, to get insights into the mechanism of action of these types of molecules: the presence of the Pt complex stabilized soluble β-structures of the Aβ_21–40_ peptide.^44^ Some of us also designed new pentacoordinate glycoconjugate platinum(II) complexes that can act as anticancer compounds.^45^ Sugar ligands were introduced aiming to enhance their biocompatibility, aqueous solubility, and recognition by cancer cells through the “Warburg effect”.^46,47^ The coordinative saturation is also an important stereoelectronic requisite that improves their general stability. In particular, the peracetylated NHC complex **1Pt** in Figure 1, prepared along with its deprotected counterpart **1Pt**_**dep**_, showed high activity and selectivity toward a panel of cell lines.^48^

Here, we present investigations focused on the ability of the two complexes to act as effective inhibitors of aggregation of Aβ peptides reported in Table 1. The ability of **1Pt**_**dep**_ to inhibit the aggregation of Aβ peptides was confirmed *via* a range of spectroscopic and biophysical techniques.

## Results and Discussion

### Effects of Pt Complexes on the Thioflavin T (ThT) Assay of Aβ_21–40_ and Aβ_25–35_

**Figure 2:**
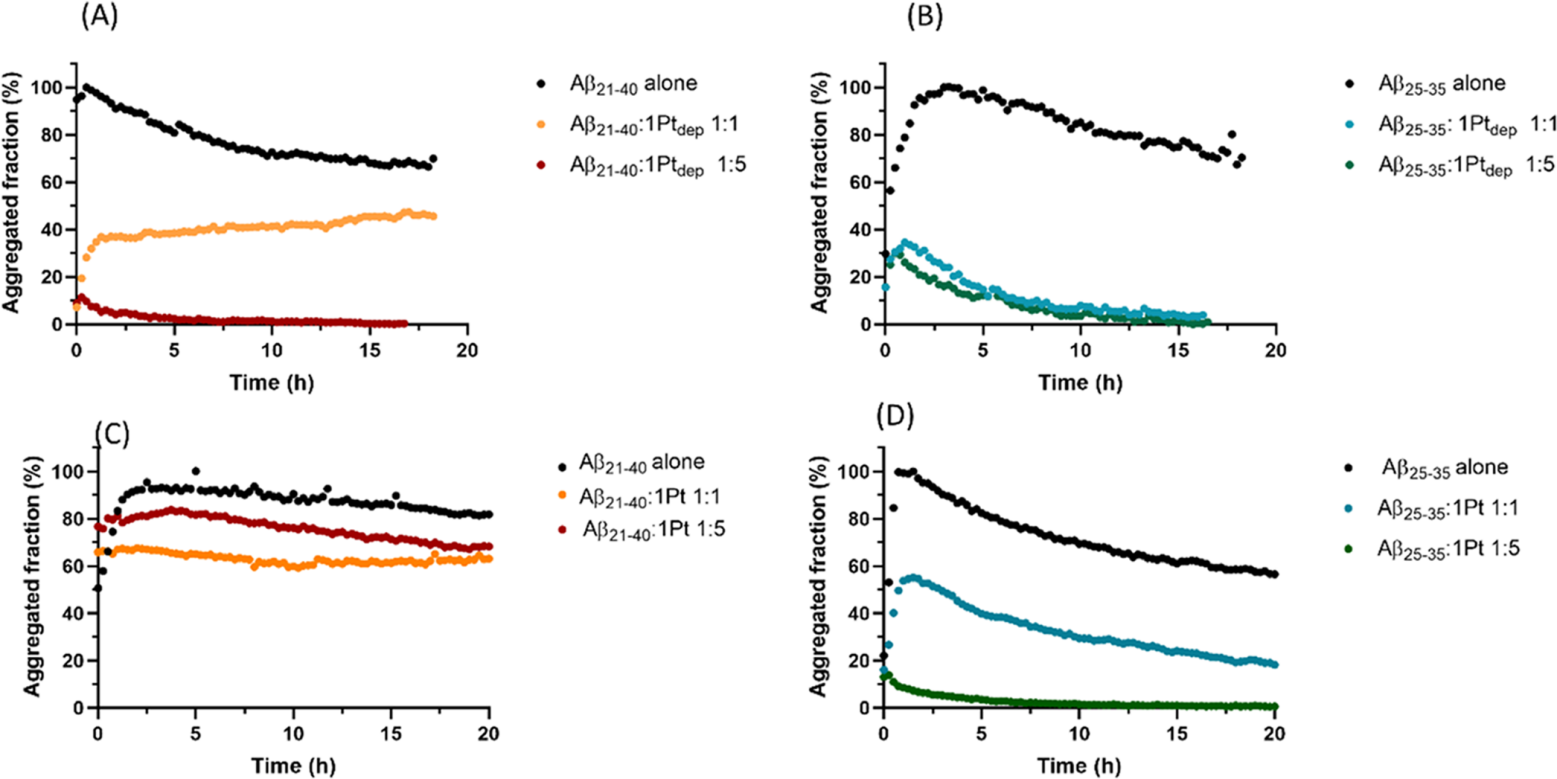
Time course of ThT fluorescence emission intensity of (A, C) Aβ_21–40_ and (B, D) Aβ_25–35_, alone and upon incubation with (A, B) **1Pt_dep_** or (C, D) **1Pt** at indicated peptide-to-metal molar ratios. Values are the average of two measurements.

Spectroscopic investigations provided evidence of the ability of both **1Pt** and **1Pt_dep_** to modulate the amyloid aggregation of Aβ-derived peptides. Initially, ThT was employed as a typical amyloid dye, which is able to bind to amyloid prefibrils, inducing a strong fluorescence signal at ∼482 nm when excited at 440 nm.^49,50^ The overlays of ThT fluorescence emission profiles of the investigated peptides in the absence and presence of **1Pt** and **1Pt_dep_** over time are reported in Figure 2.

Aβ peptide aggregation was investigated in the presence of metal complexes at peptide-to-metal molar ratios of 1:1 and 1:5. A reduction of aggregation in the presence of metal compounds is observed for almost all samples; fluorescence quenching is more evident in the case of Aβ_25–35_. Comparing the effects of Pt complexes on the Aβ_21–40_ aggregation, **1Pt_dep_** exhibits greater suppressive effects (Figure 2A) with respect to **1Pt** (Figure 2C). **1Pt_dep_** decreases aggregation of 60 and 90% at 1:1 and 1:5 peptide-to-metal molar ratios, respectively. Under similar experimental conditions (with the addition of dimethyl sulfoxide (DMSO), 2% (v/v), required for dissolution of the compound), the reduction of aggregation is less evident when the peptide is treated with **1Pt**: it is about 35 and 20% at 1:1 and 1:5 peptide-to-metal compound molar ratios, respectively (Figure 2C). The differences between the behavior of the two Pt complexes are more evident when the ThT profiles of Aβ_25–35_ are compared: when the peptide is incubated with **1Pt_dep_**, the reduction of the aggregated fraction (70%) is essentially independent of the equivalents of the complex that were used (Figure 2B); on the contrary, when the peptide is treated with **1Pt**, the behavior is similar to that observed in the experiments carried out with Aβ_21–40._ The suppressive effect of Aβ_25–35_ aggregation exerted by **1Pt** is less significant than that of **1Pt_dep_**: the level of aggregation inhibition is comparable to that exhibited by **1Pt_dep_** only when the peptide-to-metal compound molar ratio is 1:5 (Figure 2D). Noticeably, a preliminary experiment employing the entire Aβ_1–42_ sequence as aggregating polypeptide,^51^ reported in Figure S1, confirmed the ability of **1Pt_dep_** to inhibit amyloid aggregation, at a 1:5 Aβ_1–42_/**1Pt_dep_** molar ratio. Future experiments will detail the different involvements of N- and C-terminal regions in this inhibitory process.

### Electrospray Ionization Mass Spectrometry (ESI-MS) Analysis of Adducts between Aβ_21–40_, Aβ_25–35_, and Pt(II) Complexes

**Figure 3:**
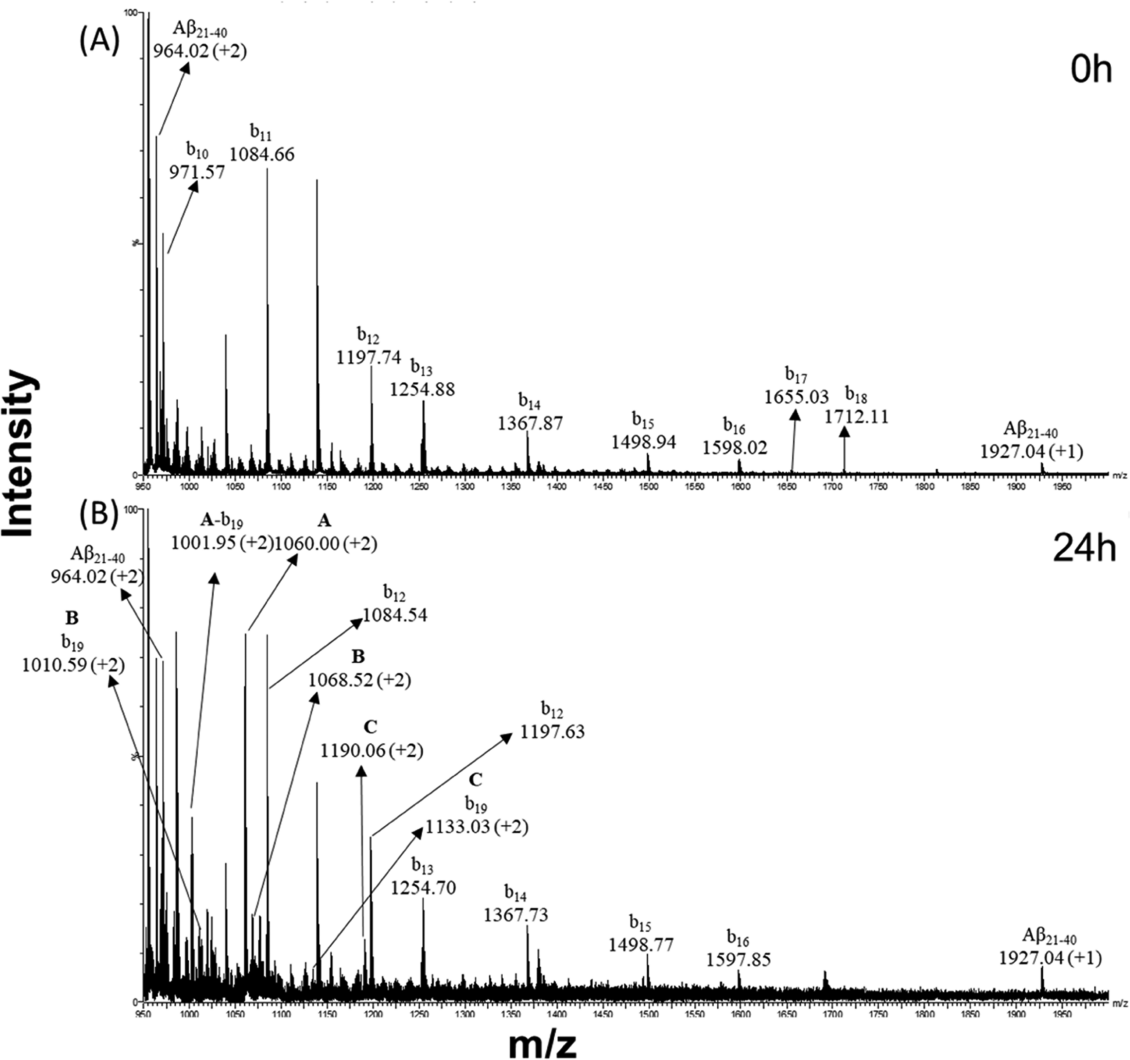
ESI-MS spectra of the Aβ_21–40_ peptide incubated with **1Pt_dep_** for (A) 0 h and (B) 24 h. Signals from ion fragmentation are also reported.

**Figure 4:**
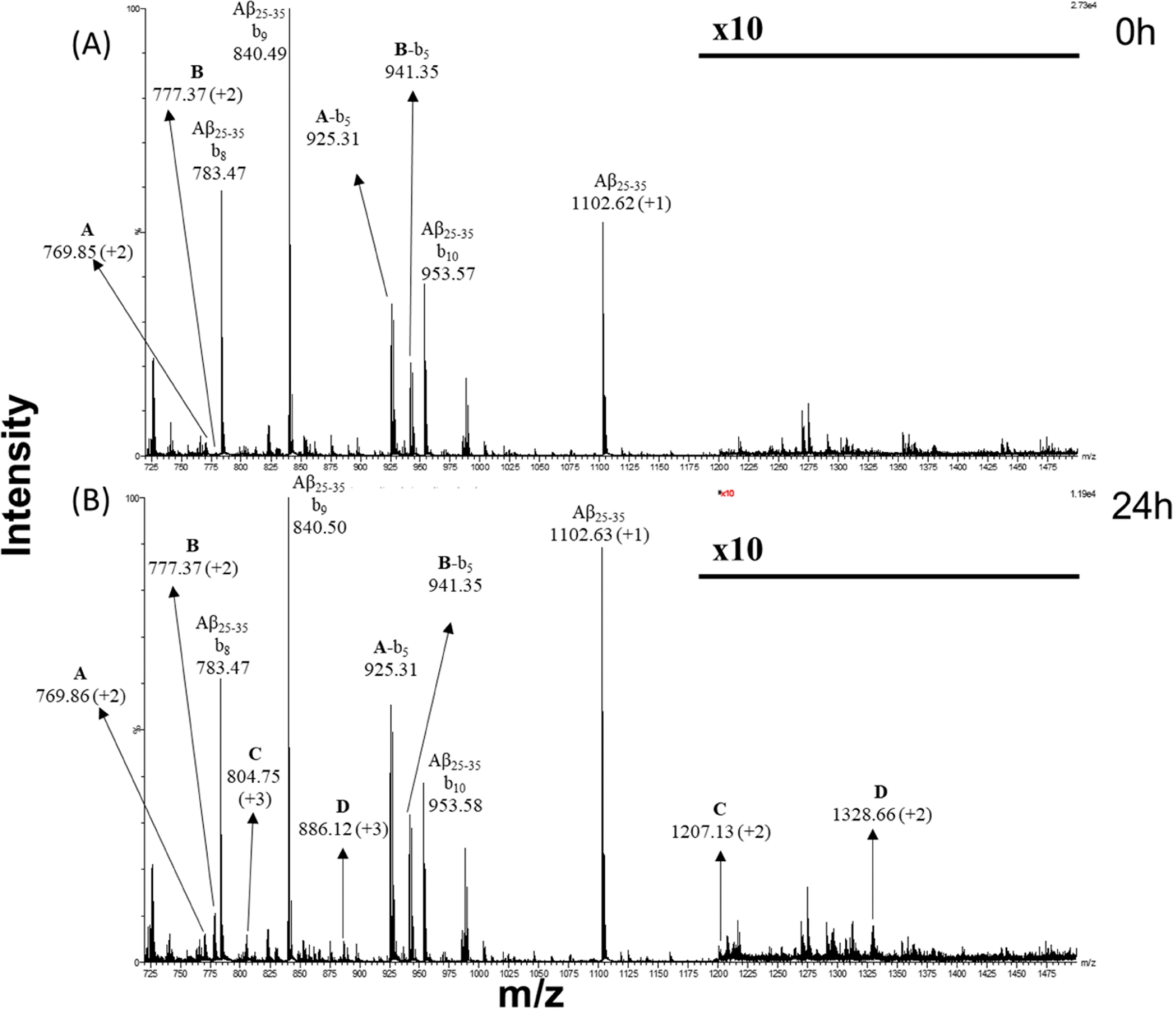
ESI-MS spectra of the Aβ_25–35_ peptide incubated with **1Pt_dep_** for (A) 0 h and (B) 24 h. Signals from ion fragmentation are also reported. The *m*/*z* range between 1200 and 1500 is 10 times magnified.

Aβ_21–40_ and Aβ_25–35_ were incubated with Pt compounds (at 1:5 peptide-to-metal complex molar ratio) and the samples were analyzed by electrospray ionization mass spectrometry (ESI-MS); the peptides alone were analyzed as references. Signals of the b series for both peptides were generated from spontaneous in-source fragmentation events (Figure S2A,B). The spectra (Figures 3 and 4) of samples containing metal complexes were registered at two times of incubation, *t* = 0 (Figures 3A and 4A) and *t* = 24 h (Figures 3B and 4B). The species recorded in Aβ_21–40_ and Aβ_25–35_ spectra are summarized in Tables 2 and 3, respectively.

**Table 2:** Results of ESI-MS of the Aβ_21–40_ Peptide Incubated for 0 and 24 h with **1Pt_dep_**^a^

component	experimental *m*/*z*, (charge state)	experimental monoisotopic mass (Da)	theoretical monoisotopic mass (Da)	Aβ_21–40_-Pt adducts	time (h)
	964.02 (+2)	1926.03 ± 0.01	1926.01	Aβ_21–40_	
	1927.04 (+1)				
A	1060.00 (+2)	2118.00	2121.09	Aβ_21–40_ + Pt(II)	24
B	1068.01 (+2)	2134.02	2136.12	Aβ_21–40_ + (Pt + Me)	24
C	1190.06 (+2)	2378.12	2379.36	Aβ_21–40_ + (Pt + sugar + Me)	24

a The *m*/*z* experimental values, experimental and theoretical monoisotopic molecular weights (MWs), and relative species are reported. In the “component” column, the labels of species in Figure 3 are reported. Me: methyl ligand; sugar: glucosyl ligand.

**Table 3:** Results of ESI-MS of the Aβ_25–35_ Peptide Incubated for 0 and 24 h with **1Pt**_**dep**_^a^

component	experimental *m*/*z*, (charge state)	experimental monoisotopic mass (Da)	theoretical monoisotopic mass (Da)	Aβ_25–35_-Pt adducts	time (h)
	1102.63 (+1)	1101.63	1100.58	Aβ_25–35_	
A	769.86 (+2)	1537.72	1538.91	Aβ_25–35_ + (Pt + sugar)	0, 24
B	777.37 (+2)	1552.75	1553.94	Aβ_25–35_ + (Pt + sugar + Me)	0, 24
C	804.75 (+3)	2411.73 ± 0.49	2411.28	2·(Aβ_25–35_) + (Pt + Me)	24
	1207.13 (+2)				
D	886.12 (+3),	2655.49 ± 0.25	2654.52	2·(Aβ_25–35_) + (Pt + sugar + Me)	24
	1328.66 (+2)				

a The *m*/*z* experimental values, experimental and theoretical monoisotopic molecular weights, and the relative species are reported. In the component column, the labels of species in Figure 4 are reported. Me: methyl ligand; sugar: glucosyl ligand.

**1Pt_dep_** appears to be able to bind to both peptides by substituting the equatorial 2,9-dimethyl-1,10-phenanthroline (Dmphen) and ethylene ligands, as demonstrated by the presence of the peak at *m*/*z* 1190.06 (Table 2, C component MW = 2378.12 Da) for Aβ_21–40_ (Figure 3B) and the peak at *m*/*z* 777.37 (Table 3, B component MW = 1552.75 Da) for Aβ_25–35_ (Figure 4). Moreover, each peptide also showed the ability to form adducts where the metal complex lacks the glucosyl axial ligand, as revealed by the presence of the species showing molecular weights of 2134.02 Da (component B in Table 2) and 2411.73 Da (component C in Table 3) and corresponding to the adducts generated by Aβ_21–40_ and Aβ_25–35_. In addition to these common features, several relevant differences emerge from the analysis of the spectra: the first difference is regarding the kinetics of adduct formation. Indeed, contrary to that found for Aβ_21–40_, Aβ_25–35_ peptide spectra (Figure 4A) show the formation of adducts at the beginning of incubation (*t* = 0). Peaks due to these adducts were assigned to Aβ_25–35_ bound to the **1Pt_dep_** fragment carrying solely the glucosyl (component A, *m*/*z* = 769.86) or both the axial ligands (component B, *m*/*z* = 777.37). Moreover, the amount of both adducts increases over time. Aβ_21–40_ showed the formation of the adduct containing glucosyl and methyl ligands only after 24 h (component C, *m*/*z* = 1190.06, Figure 3B, Table 2).

At the longest time, an additional distinguishing feature of Aβ_25–35_ samples was further detected: the occurrence of species with MWs 2411.73 ± 0.49 and 2655.49 ± 0.25 Da (components C and D, respectively, Table 3), which were both attributed to the formation of adducts with a 2:1 peptide/metal complex stoichiometry. Component D (2655.49 Da) was attributed to the adduct containing **1Pt_dep_** lacking the equatorial ligands and carrying two Aβ_25–35_ chains bound in a monodentate mode, while component C, showing 2411.73 Da as MW, consisted of the same species lacking also the axial sugar moiety. The simultaneous binding of two Aβ_25–35_ sequences is not encountered in Aβ_21–40_ samples. Conversely, only Aβ_21–40_ exhibited an adduct with naked Pt(II) ions as indicated by the presence of component A (*m*/*z* = 1060.00, Figure 3B, Table 2).

The analysis of the b series in the spectra of the peptide with **1Pt_dep_** provided insights into the peptide fragments mainly involved in the adduct formation. In the spectra of Aβ_25–35_ with **1Pt_dep_** (Figure 4), the monocharged signals at *m*/*z* 925.31 and 941.35 were due to the b_5_ element obtained by Aβ_25–35_ + (Pt + sugar) and Aβ_25–35_ + (Pt + sugar + Me) fragmentation, respectively.

The presence of the b series fragments suggested that the N-terminal stretch of the peptide is mainly responsible for the binding. Similarly, the b series signals were encountered also in the spectra of Aβ_21–40_ adducts. The doubly charged signals at *m*/*z* 1001.95, 1010.59, and 1133.03 (Figure 3B) are in accordance with the b_19_ signal of Aβ_21–40_ bound to naked Pt(II), Pt + Me, and Pt + sugar + Me fragments, as previously reported.^44^

ESI-MS analysis was also carried out by incubating β-peptides with the **1Pt** complex. In this case, no peaks deriving from adducts were detected over the time for Aβ_21–40_ (Figure S3). A peak was found only for Aβ_25–35_, in the spectral background, consistent with a 2:1 stoichiometry and compatible with an adduct of the Pt(II) complex with both the axial ligands (Figure S4). These findings confirm the results already described by Annunziata et al.:^48^ small variations in terms of the ligand structure, such as the presence of protecting groups, are able to substantially modify the binding capacity of a metal compound toward the same biomolecule. On the basis of these results, we further investigated only the ability of **1Pt_dep_** to modulate the amyloid aggregation of Aβ peptides.

### Spectroscopic Investigations of Adducts with **1Pt_dep_**

**Figure 5:**
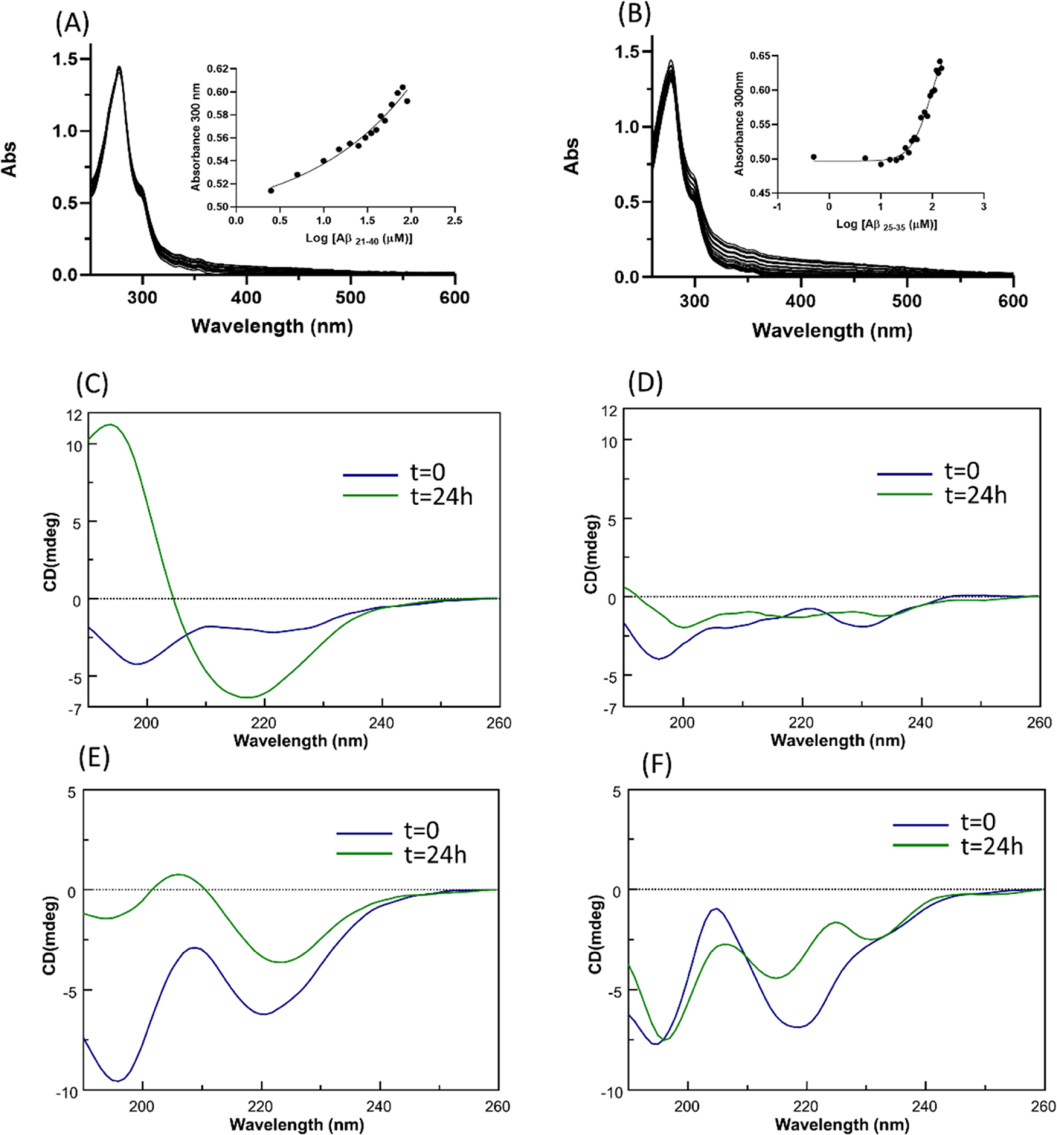
Spectroscopic investigations of the adducts of Aβ_21–40_ and Aβ_25–35_ with **1Pt**_**dep**_. Upper panel: absorption spectra of **1Pt**_**dep**_ upon the addition of increasing amounts of (A) Aβ_25–35_ and (B) Aβ_21–40_. As insets, UV intensities at indicated wavelengths *vs* log of Aβ peptide concentrations. Lower panel: overlay of circular dichroism (CD) spectra of Aβ_21–40_ (C) alone and (D) incubated with **1Pt**_**dep**_ at the 1:5 peptide/Pt(II) molar ratio and Aβ_25–35_ (E) alone and (F) incubated with **1Pt**_**dep**_ at a 1:5 peptide/Pt(II) molar ratio.

Ultraviolet–visible (UV–vis) absorption spectroscopy was employed to detect potential variations of the ligand field of **1Pt_dep_** induced by the presence of amyloid peptides. In agreement with literature studies on Pt(II)–diimine complexes,^52,53^ the spectra are characterized by the presence of the π → π* intraligand and Pt(5d) → π* metal-to-ligand charge transfer (MLCT) transitions in the 200–400 nm region. As reported in Figure 5 upper panel, an enhancement of absorbances upon increasing the amounts of both Aβ_25–35_ (Figure 5A) and Aβ_21–40_ (Figure 5B) is observable: this suggests that a mechanism of substitution of ligands around the Pt center occurred. This titration, in the case of Aβ_25–35_, allowed us to estimate EC_50_ = 93.7 ± 0.2 μM, through the fitting of absorbance values at 330 nm (inset of Figure 5A). This value is comparable to that observed in other studies of metal complex/amyloid peptide systems.^41,44^ Data fitting did not converge in the case of Aβ_21–40_, hampering the evaluation of the EC_50_ value for this peptide (inset of Figure 5B).

To evaluate if the presence of **1Pt_dep_** could have effects on the conformation of Aβ peptides, we registered CD spectra of freshly prepared samples and of the peptides incubated for 24 h with the metal compound. Spectra are reported in Figure 5. At *t* = 0, Aβ_21–40_ presented a substantial random coil profile, while Aβ_25–35_ presented a mixture of random coil and β-sheet signals;^54^ after 24 h, for both sequences, a clear conformational transition toward β-structures occurred (Figure 5C,E), as often observed during amyloid aggregation.^44,55^ Spectra registered in the presence of **1Pt_dep_**, which exhibits a nonnull Cotton effect (Figure S5), at two different times, indicate the absence of the minimum at 220 nm typical of β-sheet structures (Figure 5D,F). This finding suggests that the presence of the Pt complex causes the inhibition of the conformational transition that preludes fibrillization.

### NMR Investigations

To gain further structural insights on the interaction between **1Pt**_**dep**_ and Aβ peptides, NMR studies were carried out. We focused on Aβ_25–35_ since its sequence is more influenced by the presence of the **1Pt**_**dep**_ complex than Aβ_21–40_.

**Figure 6:**
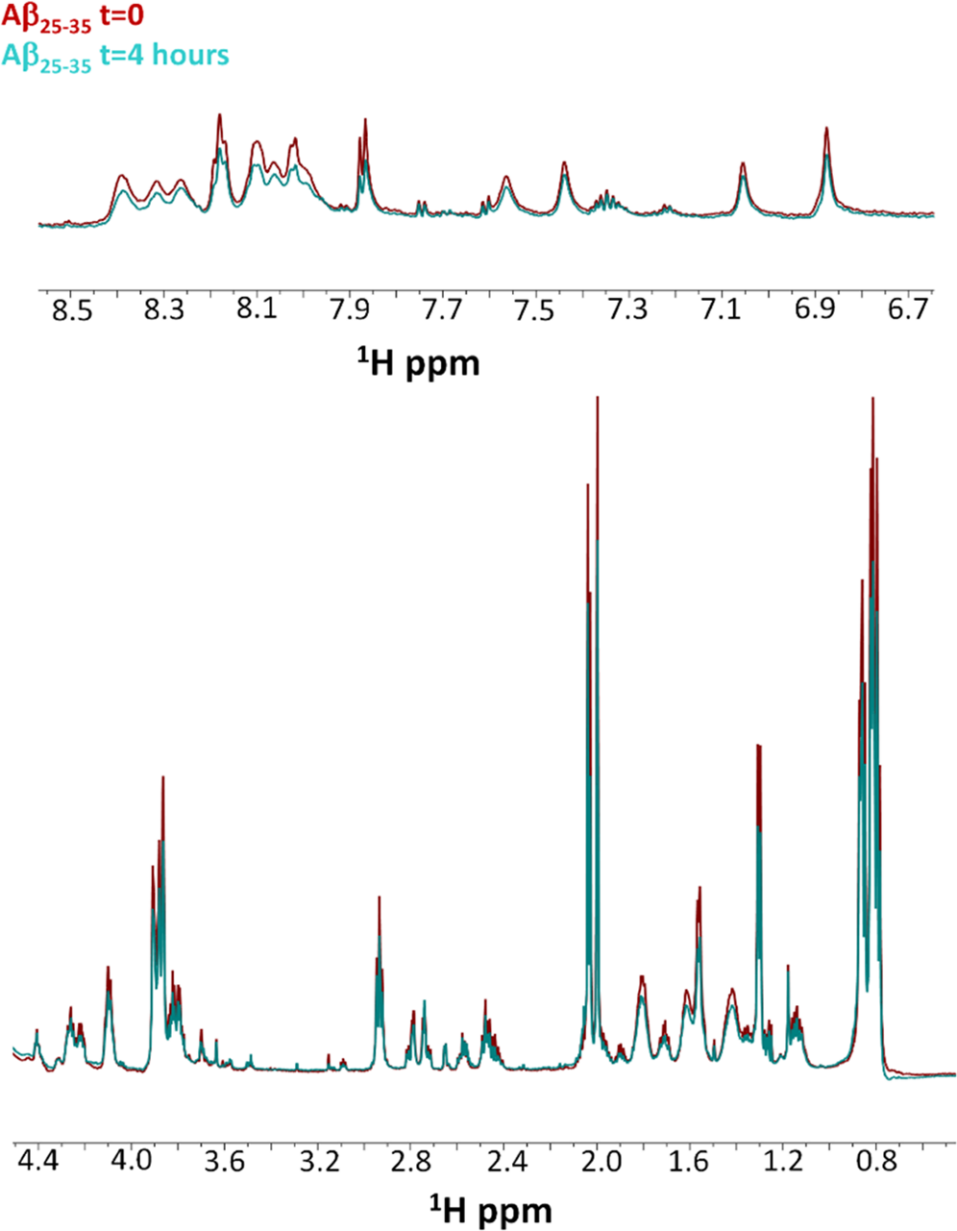
Comparison of 1D [^1^H] spectra of Aβ_25–35_ alone at *t* = 0 (red) and 4 h (cyan). The H_N_ region is shown in the upper panel, the H_α_ and side chain proton region is reported in the lower panel.

We first run one-dimensional (1D) [^1^H] spectra, reported in Figure 6, of Aβ_25–35_ alone at *t* = 0 and 4 h from a freshly prepared sample. Spectra at *t* = 0 and 4 h present rather sharp signals, except for the solvent-exposed H_N_ peaks (Figure 6 upper panel), indicating the presence of species with small molecular weights and a disordered organization. This is not surprising as large oligomers and protofibrils cannot be observed by solution NMR due to fast relaxation, while disaggregated and/or small oligomers are NMR visible.^56,57^ After 4 h, a slight decrease of signal intensity is observed (Figure 6). This suggests that large aggregates are formed, although most of the peptide remains in the disaggregated and/or in small oligomer forms (Figure 6).

**Figure 7:**
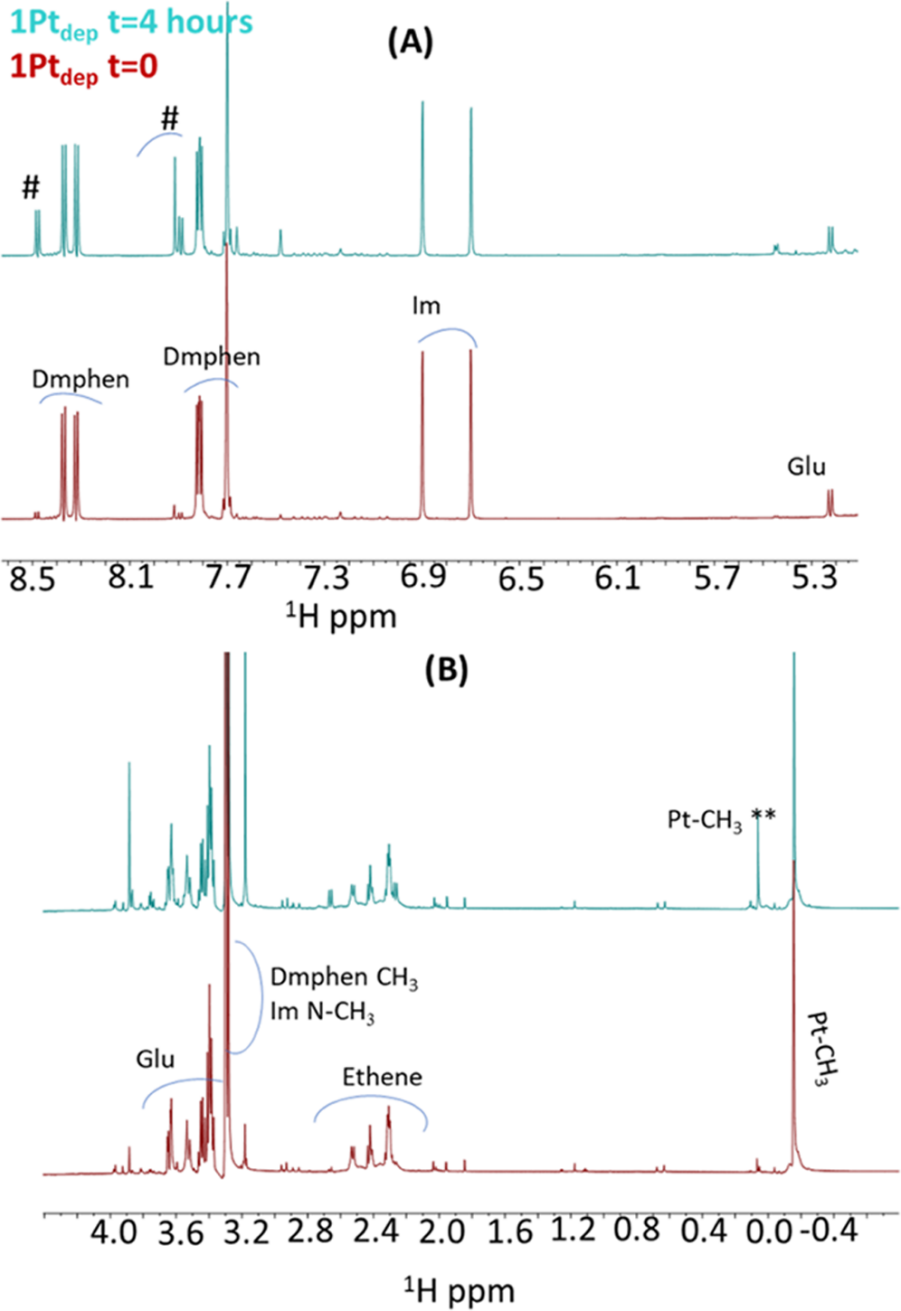
Comparison of 1D [^1^H] spectra of **1Pt**_**dep**_ at *t* = 0 (red) and 4 h (cyan). Intervals of chemical shift: (A) 5.3–8.5 and (B) −0.4 to 4.0 ppm. Assignment of Pt ligands is reported at *t* = 0. Dmphen: 2,9-dimethyl-1,10-phenanthroline, Im: imidazole derivative, Glu: glucosyl unit. On the top cyan spectrum, peaks arising from free Dmphen are indicated by #, while ** refers to Pt-CH_3_ in a square-planar complex.^48^

A two-dimensional (2D) [^1^H, ^1^H] total correlation spectroscopy (TOCSY) spectrum reported in Figure S6 allows us to distinguish side chain protons of different residues of Aβ_25–35_. 1D [^1^H] NMR spectra were also recorded for **1Pt**_**dep**_ alone at *t* = 0 and 4 h and are reported in Figure 7.

After 4 h, many changes occur in the spectrum: additional signals appear, and the peaks of the main compound decrease in intensity. New peaks are assigned to free ligands that are released from the metal coordination sphere. Indeed, previous ^1^H NMR studies of the **1Pt** analogue compound conducted in DMSO-*d*_6_ indicated that both Dmphen and ethylene could be displaced by solvent molecules leading, over time, to square-planar species.^48^ In detail, the signal close to 0.0 ppm is due to Pt-CH_3_ in a square-planar geometry, supporting the coexistence of square-planar along with the bipyramidal trigonal geometry that, however, is still predominant after 4 h^48^ (Figure 7B).

**Figure 8:**
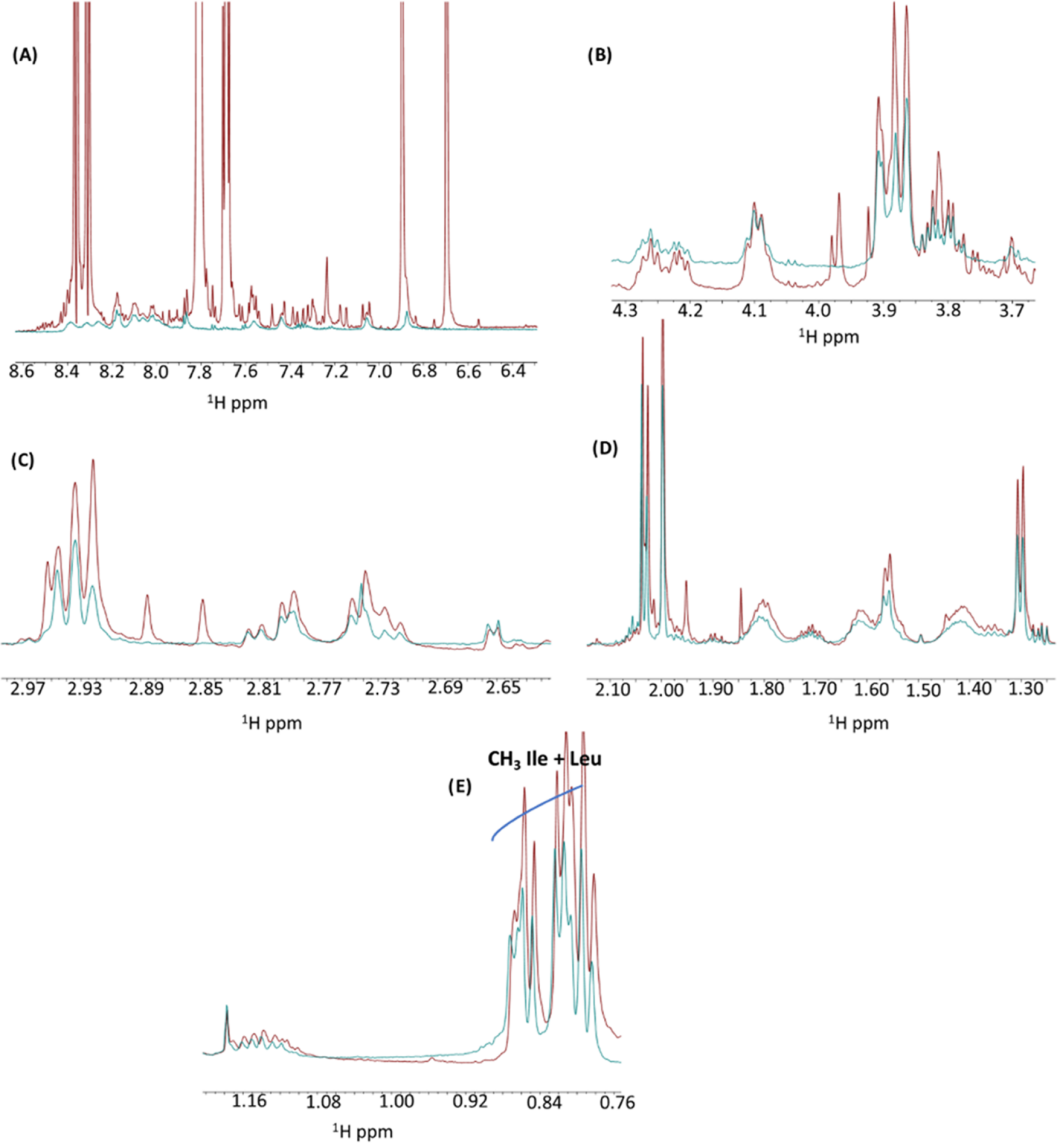
Overlay of 1D [^1^H] spectra of **1Pt**_**dep**_ + Aβ_25–35_ (red) and Aβ_25–35_ alone (cyan) at *t* = 4 h. Different chemical shift regions are reported in each panel.

1D [^1^H] spectra were also acquired for Aβ_25–35_ in the presence of **1Pt**_**dep**_ (1:5 peptide/metal molar ratio). Spectra recorded at *t* = 0 and 4 h appear quite similar, indicating that no time effects are observable under NMR conditions (Figure S7). Interestingly, in the comparison of 1D [^1^H] spectra of **1Pt**_**dep**_ at *t* = 0 and of **1Pt**_**dep**_ + Aβ_25–35_ at either *t* = 0 or 4 h, no relevant changes can be observed, clearly indicating a mutual influence between the complex and the peptide. Interestingly, it can be noted that the presence of Aβ_25–35_ stabilizes the bipyramidal trigonal geometry of the metal compound since ^1^H signals from the **1Pt**_**dep**_ spectrum recorded at *t* = 0 can be overlayed with those present in the spectrum of **1Pt**_**dep**_ + Aβ_25–35_ at *t* = 4 h if one excludes minor chemical shift changes (Figure S8). By comparing 1D [^1^H] spectra of Aβ_25–35_ alone and **1Pt**_**dep**_ + Aβ_25–35_ at *t* = 4 h, a few chemical shift changes could be noticed (Figure 8): these variations do not affect H_N_ (Figure 8A) and H_α_ (Figure 8B) protons but concern mainly with CH_2_ (Figure 8C,D) and CH_3_ (Figure 8E) side chain protons, except for the serine residue that seems unaffected (Figure 8B). The chemical shift changes indicate some conformational variations induced in the Aβ_25–35_ peptide by the presence of **1Pt**_**dep**_.

### Structural Analysis of Amyloid Fibers of Aβ-Derived Peptides in the Presence of **1Pt_dep_**

**Figure 9:**
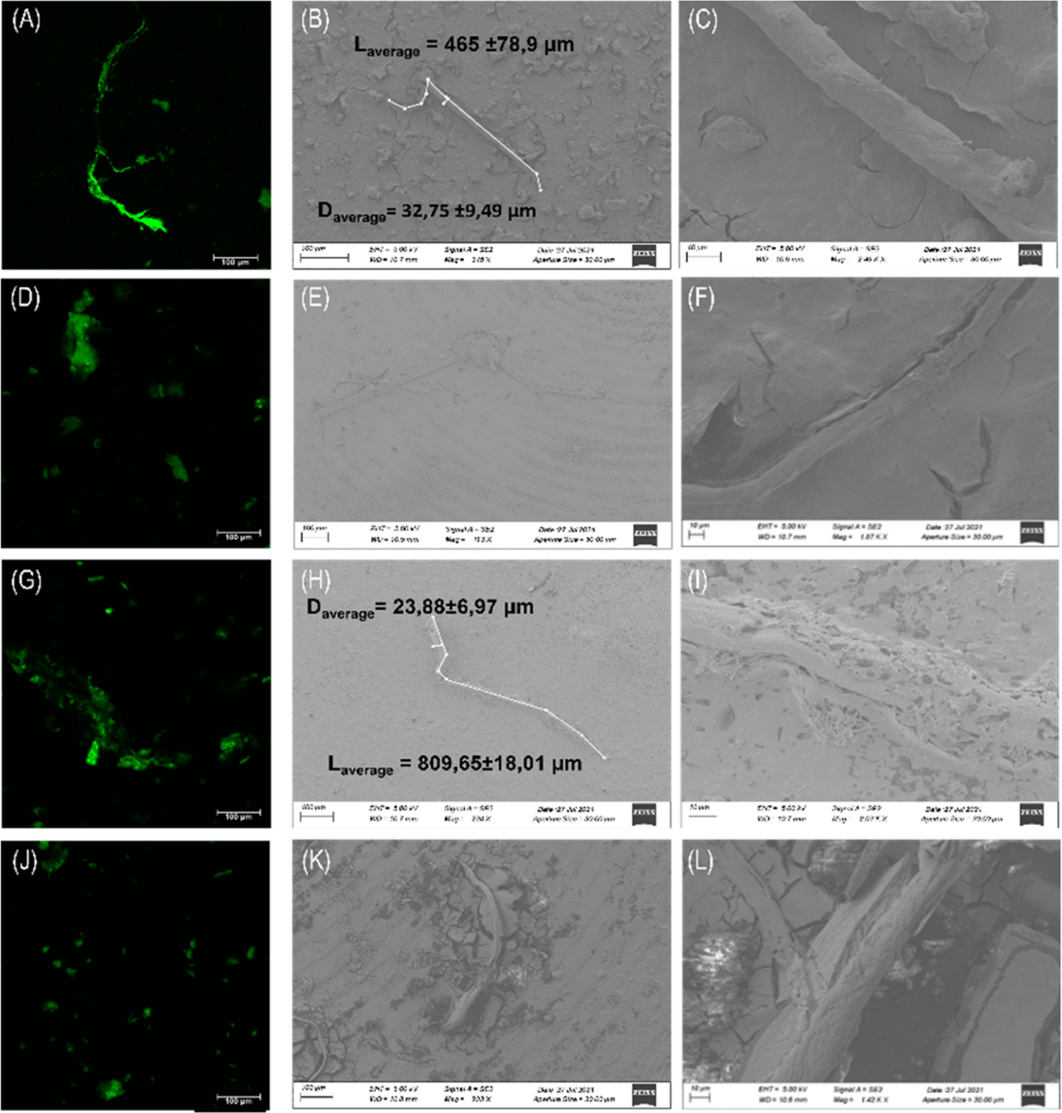
Microscopic studies of Aβ peptides in the absence and presence of **1Pt**_**dep**_. Confocal microscopy of ThT incubated systems: (A, G) Aβ_21–40_ and Aβ_25–35_ alone, (D) Aβ_21–40_:**1Pt**_**dep**_, (J) Aβ_25–35_:**1Pt**_**dep**_ both at a 1:5 ratio (λ_exc_ = 440 nm and λ_emiss_ between 460 and 600 nm). SEM micrographs of (B, C) Aβ_21–40_ and (E, F) Aβ_21–40_:**1Pt**_**dep**_. (H, I) Aβ_25–35_ and (K, L) Aβ_25–35_:**1Pt**_**dep**_ at 1:5, at (B, E, H, K) 100 μm and (C, F, I, L) 10 μm.

To get insights into the morphology of the aggregates derived from Aβ peptides in the presence and absence of **1Pt**_**dep**_, confocal and scanning electron microscopies (SEMs) were used. Confocal analysis of Aβ peptides alone presents typical amyloid fiber shapes (Figure 9A,G), while the presence of the metal complex at a 1:5 metal complex-to-peptide molar ratio determines the suppression of the fiber, favoring the formation of amorphous aggregates (Figure 9D,J). SEM experiments corroborate these results: micrographs registered at different magnifications (reported in Figure 9) delight the presence of well-structured fibers with an average diameter of 3.3 ± 1.0 × 10 μm and a length of 4.7 ± 0.8 × 10^2^ μm for Aβ_21–40_ (Figure 9B,C) and a diameter of 24 ± 7 μm and a length of 8.1 ± 0.2 × 10^2^ μm for Aβ_25–35_ (Figure 9H,I). The presence of **1Pt**_**dep**_ perturbs microstructure formation in this case as well: the microstructures appear immersed in the stub matrix, not a well defined and faintly visible event at high magnification values (Figure 9E,F,K,L).

## Experimental Section

### Reagent Syntheses

**1Pt** and **1Pt**_**dep**_(48) and Aβ_21–40_ and Aβ_25–35_ peptides^58^ were synthesized as already reported, synthetic Aβ_1–42_ was purchased from Abcam, and related sequences are reported in Table 1. After purification, they were treated with 1,1,1,3,3,3-hexafluoro-2-propanol (HFIP) and then stored at −20 °C until use.

### Fluorescence Assays

ThT fluorescence assays were performed at 25 °C, employing a peptide concentration of 100 μM for Aβ_21–40_ and 200 μM for Aβ_25–35_ in 10 mM phosphate buffer at pH 7.4, using a ThT final concentration of 50 μM, at different ratios with Pt(II) complexes (stock solutions 1 mM in water for **1Pt**_**dep**_ and in 100% DMSO for both complexes). ThT experiments that have been carried out with **1Pt** were acquired in solutions containing DMSO at 2% (v/v). In this solvent, **1Pt** is stable, as suggested by UV–vis absorption spectra collected as a function of time and reported in Figure S9. To correctly compare the time course fluorescence intensities of the peptides in the presence of **1Pt** and **1Pt**_**dep**_, percentages of aggregated fractions are reported. Fluorescence was measured using a Jasco FP 8300 fluorescence spectrofluorometer in a 1 cm cuvette under magnetic stirring. The excitation wavelength was 440 nm and the emission wavelength was between 450 and 600 nm. A scanning speed of 100 nm/min was used. Spectra were recorded every 15 min at the indicated times and assays were performed in duplicates. The fluorescence intensity peak was determined at 482 nm.

### Circular Dichroism

CD spectra of Aβ_21–40_ (50 μM) and Aβ_25–35_ (100 μM) in 10 mM phosphate buffer, alone or at a 1:2.5 peptide-to-metal complex molar ratio with **1Pt**_**dep**_, were registered on a Jasco J-815 spectropolarimeter (JASCO, Tokyo, Japan), in a 0.1 cm cuvette.

### UV–Vis Absorption Spectroscopy

UV–vis spectra of **1Pt**_**dep**_ at increasing amounts of Aβ peptides were registered on a Nanodrop 2000c spectrophotometer (Thermo Scientific, Milan). The complex concentration was fixed at 50 μM. Peptides were added through the incorporation of 2.0 μL of peptide stock solution (500 μM) in water, kept at 0 °C. Spectra were recorded in the range of 260–600 nm after the peptide addition and 2 min under stirring. Upon addition, the metal complex-to-peptide molar ratio was 1:3 for both peptides. The estimation of the EC_50_ value was obtained from the nonlinear regression using the “dose–response stimulation equation” of GraphPad program and employing log [inhibitor] *vs* absorbance intensities as experimental data.^59^

### ESI-MS Analysis

Solutions of Aβ_21–40_ and Aβ_25–35_ at a concentration of 50 μM in 15 mM ammonium acetate (pH = 6.8), at a 1:5 molar ratio with Pt(II) complexes, were incubated for two different time durations (0 and 24 h). The reaction mixtures were diluted 10 times with 15 mM ammonium acetate (pH = 6.8) and then analyzed using a Q-ToF Premier (Waters, Milliford, MA) mass spectrometer. For **1Pt**_**dep**_, the *m*/*z* acquisition range spanned from 900 to 2500 *m*/*z* and from 500 to 1500 *m*/*z* for Aβ_21–40_ and Aβ_25–35_. Differently, for **1Pt**, the Aβ_25–35_*m*/*z* acquisition range was changed to 500–2000 *m*/*z*.

### Scanning Electron Microscopy

SEM analysis was performed with an Ultra Plus FESEM scanning electron microscope (Zeiss, Germany). Samples (100 μL) containing Aβ_21–40_ (100 μM) and Aβ_25–35_ (200 μM), alone or mixed with **1Pt**_**dep**_ at a 1:5 ratio (10 mM phosphate buffer), were mounted on stub and gold-sputtered at 20 nm thickness using a HR208 Cressington sputter coater and analyzed at 10–15 kV with an SE2 detector, as already reported.^60,61^ Fiber diameters and length were determined by ImageJ software.

### Confocal Microscopy

Confocal microscopy analysis was performed by a Leica SP5 microscope using a HCX IRAPO L 40×/0.95 water objective, as previously reported.^62^ Aβ_21–40_ (100 μM) and Aβ_25–35_ (200 μM) alone or with **1Pt**_**dep**_ at a 1:5 ratio incubated with ThT (50 μM) (50 mM phosphate buffer) were analyzed using an excitation of 440 nm and an emission between 460 and 600 nm.

### NMR Assays

Analyzed NMR samples were (1) Aβ_25–35_ (400 μM), (2) **1Pt**_**dep**_ (2 mM), and (3) Aβ_25–35_+ **1Pt**_**dep**_ (400 μM:2 mM nominal concentration) at *t* = 0 and 4 h of aggregation. Final volumes were of 540 μL (500 μL of sodium phosphate buffer pH 7.2 and 40 μL of D_2_O (deuterium oxide, 98% D, Sigma-Aldrich, Milan, Italy). NMR spectra were recorded on a Bruker Avance 700 MHz spectrometer at 294 K. 1D [^1^H] spectra were recorded with 64 scans. In addition, a 2D [^1^H, ^1^H] TOCSY^63^ spectrum was recorded for Aβ_25–35_ with mixing time equal to 70 ms after 4 h. The 2D [^1^H, ^1^H] TOCSY spectrum was acquired with 58 scans, 128 free induction decays (FIDs) in t1, and 1024 data points in t2. Water suppression was achieved by presaturation. Spectra were processed and analyzed with TopSpin4.1.1 (Bruker, Italy). The 2D TOCSY spectrum was analyzed with NEASY^64^ included in the Cara (computer-aided resonance assignment, http://www.nmr.ch/). Chemical shifts were referenced to the residual water peak at 4.75 ppm.

### 3-[4,5-Dimethylthiazol-2-yl]-2,5-diphenyl Tetrazolium Bromide (MTT) Assay

Human SH-SY5Y neuroblastoma cells were grown in Dulbecco’s modified Eagle’s medium (DMEM) (GIBCO, Paisley, U.K.) containing 10% heat-inactivated fetal bovine serum (FBS) (GIBCO), supplemented with 2 mM l-glutamine, 50 ng/mL streptomycin, and 50 units/mL penicillin and maintained in a humidified atmosphere (5% CO_2_ at 37 °C). Once at 70–80% of confluence, cells were harvested with 0.25% trypsin (Sigma-Aldrich, St. Louis, MO). Aβ_21–40_ and Aβ_25–35_ alone or with **1Pt**_**dep**_ or **1Pt** at a 1:5 molar ratio, respectively, were incubated in 50 mM sodium phosphate buffer, pH 7.2, under stirring, and samples were taken at three different times: 0, 2, and 24 h. Peptides were added to the cells in culture media in 96-well plates at 100 μM and then incubated for 24 h at 37 °C. Cell viability was then assessed by the 3-(4,5-dimethylthiazol-2-yl)-2,5-diphenyl tetrazolium bromide (MTT) assay as previously described.^55,65^

## Conclusions

The present study well reflects our recent focus on the applicability of metal-based anticancer drugs in the field of neurodegeneration: by assuming different amyloidogenic peptides as model systems of neurodegenerative proteins, we assessed the ability of several metal complexes with different metal ions (such as Pt, Ru, and Au) and diverse geometries (square-planar and octahedral) to act as inhibitors of self-aggregation processes.^40−42,44^

Herein, we have carried out several biophysical investigations to deepen, at the molecular level, the ability of two glycoconjugate Pt(II) bipyramidal complexes (Figure 1) to modulate amyloid aggregation. For its crucial involvement in AD, we have assumed as amyloid systems two fragments of the Aβ polypeptide: Aβ_21–40_ and Aβ_25–35_.

In the first assay, ThT fluorescence of the peptides in the absence and presence of metal compounds over time has been registered. Data indicated a clear suppression of the aggregation process of both the peptides, mostly by water-soluble **1Pt**_**dep**_, with a greater effect on the inhibition of aggregation of Aβ_25–35_. ESI-MS and UV–vis absorption spectroscopy experiments outline that the inhibition of aggregation occurs through the formation of adducts between the Pt(II) bipyramidal complexes and Aβ peptides, through the insertion of peptides into the coordination sphere of the Pt center that implies the preferential release of the axial ligands, even if other exchanges can occur. ESI-MS analysis also indicated that in the case of the Aβ_25–35_/**1Pt**_**dep**_ system, the metal complex can coordinate two peptide molecules at the same time, forming an adduct with 1:2 metal/peptide stoichiometry. This adduct cannot be formed in the case of Aβ_21–40_, probably because of its longer sequence. Conversely, Aβ_25–35_ was the only sequence to provide an adduct, although of minimum intensity, with **1Pt**. This complex has a lower intrinsic ability to form adducts with the peptides, probably because of its minor capacity, when compared to its deprotected analogue, to form hydrogen bonds that could be important in the early stage of the peptide/metal complex recognition process that precedes the formation of the coordinative bond. Because of limited ability of **1Pt** to form adducts with the investigated peptides, we focused on **1Pt**_**dep**_ in further studies. From a conformational perspective, both CD and NMR experiments pointed out a deep mutual influence between the amyloidogenic peptide and **1Pt**_**dep**_; indeed, the presence of the metal complex stabilizes monomeric forms/small aggregate forms of the peptide that do not evolve, during time, toward large aggregated species. In NMR assays, we observed that also the bipyramidal geometry of **1Pt**_**dep**_ is stabilized by the presence of Aβ_25–35_. Finally, microscopy investigations confirmed all spectroscopic data showing that **1Pt**_**dep**_ suppresses the formation of amyloid fibers for both Aβ sequences. Unfortunately, the Pt complexes investigated in this study are not able to rescue the cytotoxicity induced by amyloid peptides, as reported in Figure S10. Thus, these compounds cannot be directly translated as neurodrugs, but, instead, they can be assumed as valid templates to develop more specific drugs, preferentially able to cross the brain barrier.

In conclusion, this study represents an important example of how biophysical characterization of the adducts formed upon the reaction of metallodrugs with amyloid peptides can highlight on their mechanism of aggregation inhibition and is promising for the application of analogous glycoconjugate Pt(II) bipyramidal derivatives as novel therapeutics in neurodegenerative diseases.

## Supporting Information Available

Time course of ThT fluorescence emission intensity of Aβ_1–42_, alone and upon incubation with **1Pt**_**dep**_ at the indicated peptide-to-metal molar ratio: the values are the average of two measurements (Figure S1); ESI-MS spectra of Aβ_21–40_ and Aβ_25–35_ alone (Figure S2); ESI-MS spectra of the Aβ_21–40_ peptide incubated with **1Pt** for 0 h and 24 h (Figure S3); ESI-MS spectra of the Aβ_25–35_ peptide incubated with **1Pt** for 0 h and 24 h (Figure S4); overlay of CD spectra at 0 and 24 h of **1Pt**_**dep**_ (Figure S5); 2D [1H, 1H] TOCSY spectrum of Aβ_25–35_ recorded at *t* = 4 h (Figure S6); overlay of 1D [1H] NMR spectra of **1Pt**_**dep**_ plus Aβ_25–35_ (Figure S7); comparison between 1D [^1^H] NMR spectra of **1Pt**_**dep**_ alone at *t* = 0 and **1Pt**_**dep**_ in the presence of Aβ_25–35_ at *t* = 4 h (Figure S8); time course UV–vis spectra of **1Pt** at 50 μM in 2% DMSO phosphate buffer, 10 mM, at pH 7.4 (Figure S9); survival of SH-SY5Y cells treated with Aβ_21–40_ and Aβ_25–35_ (100 μM) alone or with **1Pt**_**dep**_ or **1Pt** at the 1:5 molar ratio at three time points: 0, 2, and 24 h; cells with peptides were incubated for 24 h and then processed for the MTT test (**p* < 0.05, ***p* < 0.005, ^#^*p* < 0.001, and ^&^*p* < 0.0001 at statistical analysis of one experiment performed in triplicates) (Figure S10) (PDF)
