# Supplementary material for: Glucosyl Platinum(II) Complexes Inhibit Aggregation of the C-Terminal Region of the Aβ Peptide
Source: Inorg Chem. 2022 Feb 16;61(8):3540–52. doi: 10.1021/acs.inorgchem.1c03540 (PMC9951207; doi:10.1021/acs.inorgchem.1c03540)
Supplement: Supplementary file 1 — ic1c03540_si_001.pdf [file ic1c03540_si_001.pdf]

Supporting Information:

A glucosyl platinum(II) complex inhibits the aggregation of the C-terminal region of Aβ peptide

Sara La Manna[1], Marilisa Leone[2], Ilaria Iacobucci[3, 4], Alfonso Annuziata[3], Concetta Di Natale[5], Elena Lagreca[5], Anna Maria Malfitano[6], Francesco Ruffo[3], Antonello Merlino[3], Maria Monti [3,4,] and Daniela Marasco[1,2,]**

[1]Department of Pharmacy, University of Naples "Federico II", 80131, Naples, Italy

[2]Institute of Biostructures and Bioimaging - CNR, 80134, Naples, Italy

[3]Department of Chemical Sciences, University of Naples "Federico II", 80126, Naples, Italy

[4]CEINGE Biotecnologie Avanzate S.c.a r.l., "University of Naples Federico II", 80131, Naples, Italy

[5]Interdisciplinary Research Centre on Biomaterials (CRIB), Department of Ingegneria Chimica dei Materiali e della Produzione Industriale (DICMAPI), University "Federico II", 80125 Naples, Italy

[6]Department of Translational Medical Science, University of Naples "Federico II", 80131, Naples, Italy

Table of contents:

Figure S1: Time course of ThT fluorescence emission intensity of $A\beta_{1\text{-}42}$, alone and upon the incubation with **1Pt**$_{dep}$ at the indicated peptide to metal molar ratio. Values are the average of two measurements.

Figure S2: ESI-MS spectra of the $A\beta_{21\text{-}40}$ and $A\beta_{25\text{-}35}$ alone.

Figure S3: ESI-MS spectra of $A\beta_{21\text{-}40}$ peptide incubated with **1Pt** for 0h and 24h.

Figure S4: ESI-MS spectra of $A\beta_{25\text{-}35}$ peptide incubated with **1Pt** for 0h and 24h.

Figure S5: Overlay of CD spectra at 0 and 24 h of **1Pt**$_{dep}$.

Figure S6: 2D [^{1}H, ^{1}H] TOCSY spectrum of A$\beta_{25\text{-}35}$ recorded at t=4h.

Figure S7: Overlay of 1D [^{1}H] NMR spectra of **1Pt$_{dep}$** plus A$\beta_{25\text{-}35}$.

Figure S8: Comparison between 1D [^{1}H] NMR spectra of **1Pt$_{dep}$** alone at t=0 and **1Pt$_{dep}$** in presence of A$\beta_{25\text{-}35}$ at t=4 hours.

Figure S9: Time course UV-vis spectra of **1Pt** at 50 μM in 2% - DMSO phosphate buffer 10 mM at pH 7.4.

Figure S10: Cell survival of SH-SY5Y cells treated with A$\beta_{21\text{-}40}$ and A$\beta_{25\text{-}35}$ (100 μM) alone or with **1Pt$_{dep}$** or **1Pt** at 1:5 molar ratio at three time points: 0, 2 and 24 hours. Cells with peptides were incubated for 24h and then processed for MTT test. (*p< 0.05, **p< 0.005, #p< 0.001, &p< 0.0001 at statistical analysis of one experiment performed in triplicates).

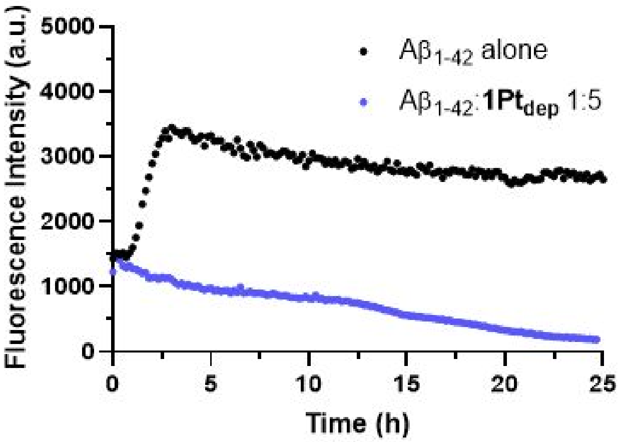

Figure S1. Time course of ThT fluorescence emission intensity of A$\beta_{1\text{-}42}$, alone and upon the incubation with **1Pt$_{dep}$** at the indicated peptide to metal molar ratio. Values are the average of two measurements.

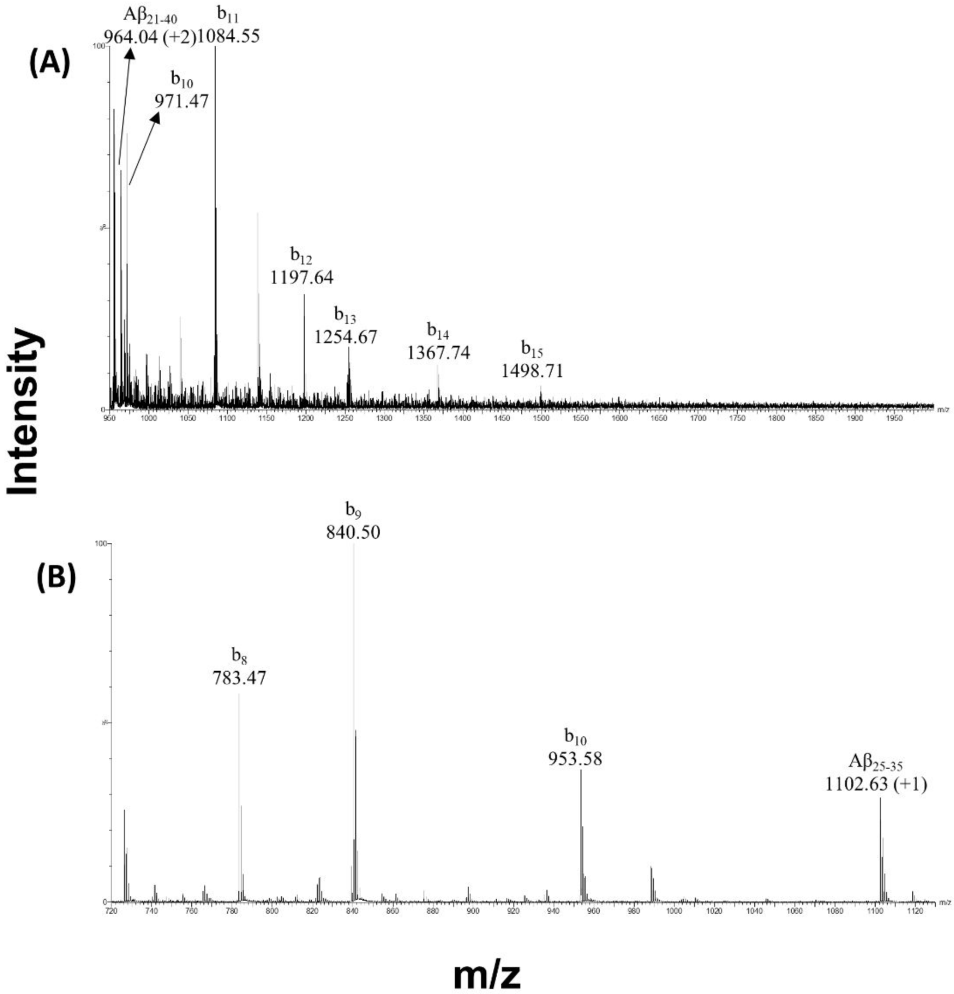

Figure S2. ESI-MS spectra of the (A) $A\beta_{21-40}$ and (B) $A\beta_{25-35}$ alone. Fragmentation ions are also reported.

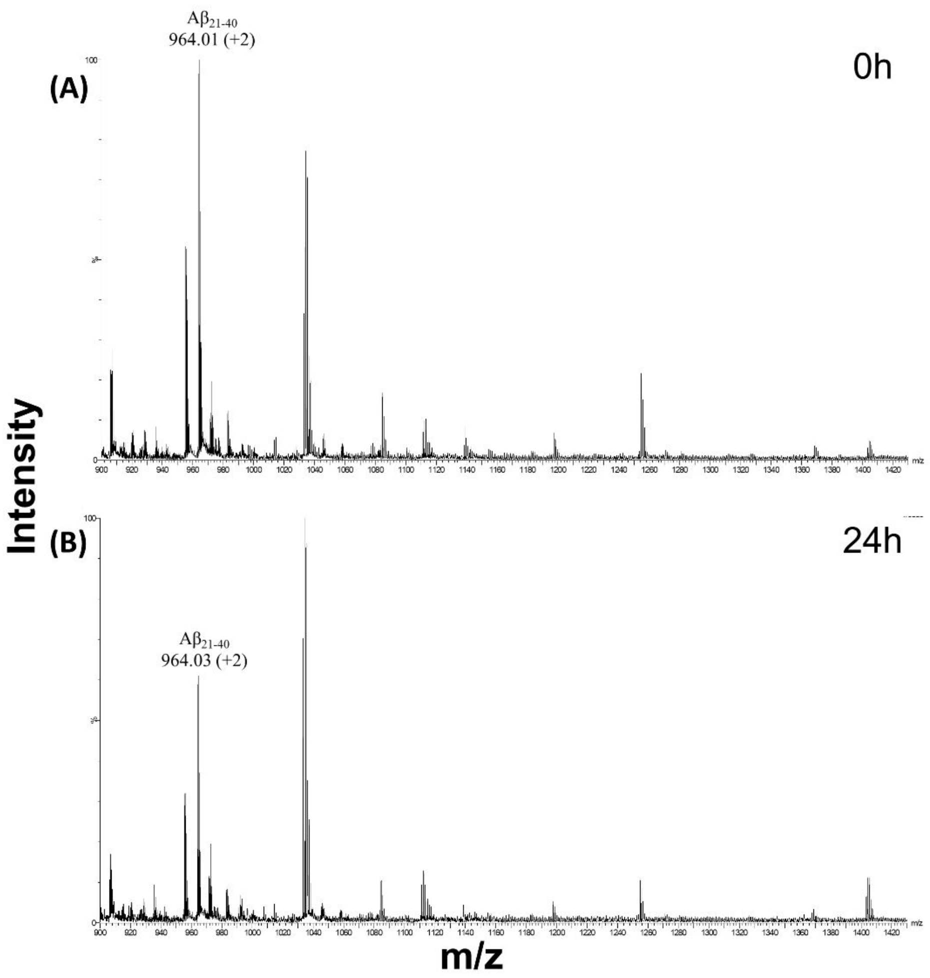

Figure S3. ESI-MS spectra of Aβ_{21-40} peptide incubated with **1Pt** for (A) 0h and (B) 24h.

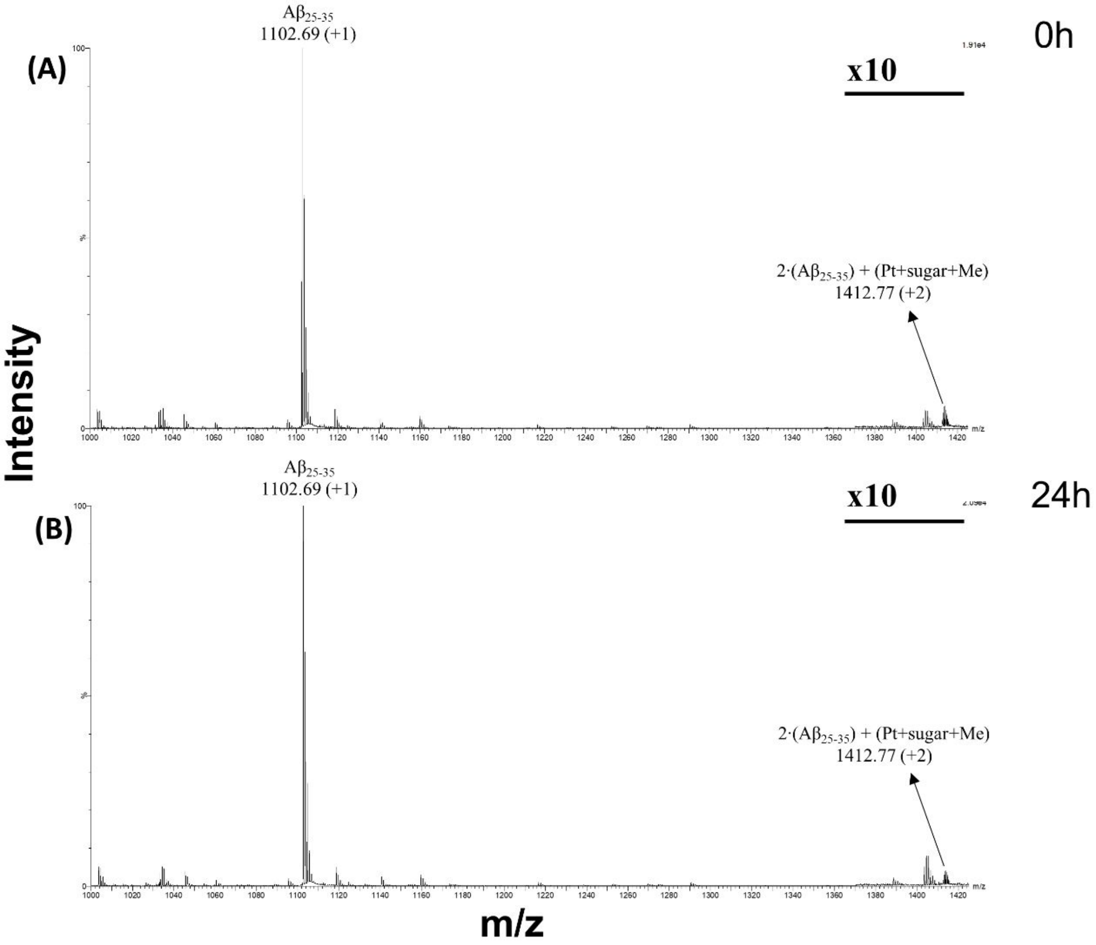

Figure S4. ESI-MS spectra of A$\beta_{25\text{-}35}$ peptide incubated with **1Pt** for (A) 0h and (B) 24h. The m/z range between 1300 and 1500 is 10 times magnified.

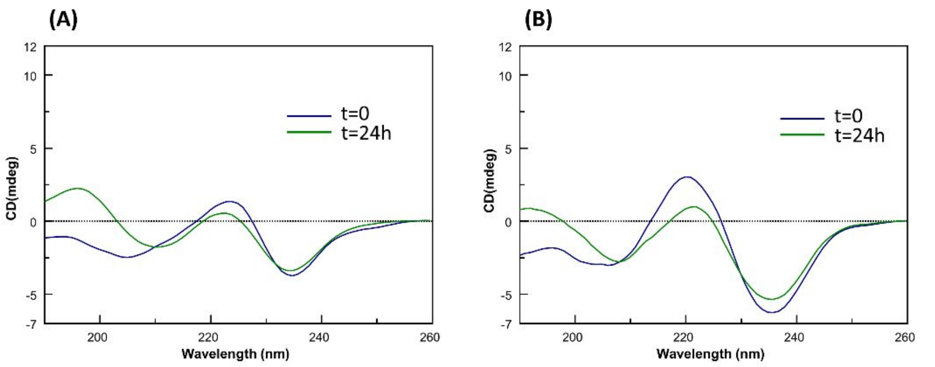

Figure S5. Overlay of CD spectra at 0 and 24 h of **$1Pt_{dep}$** at (A) 125μM and (B) 250 μM.

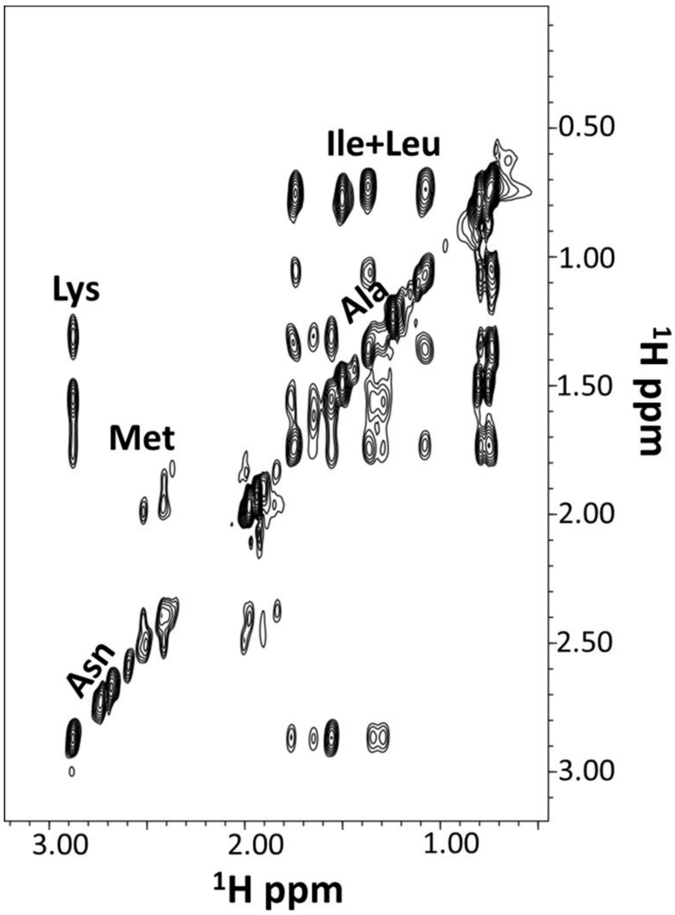

Figure S6. 2D [^{1}H, ^{1}H] TOCSY spectrum of A$\beta_{25\text{-}35}$ recorded at t=4h. Correlations arising from $_C$H$_2$ and $_C$H$_3$ side chain protons can be seen in the reported expansion.

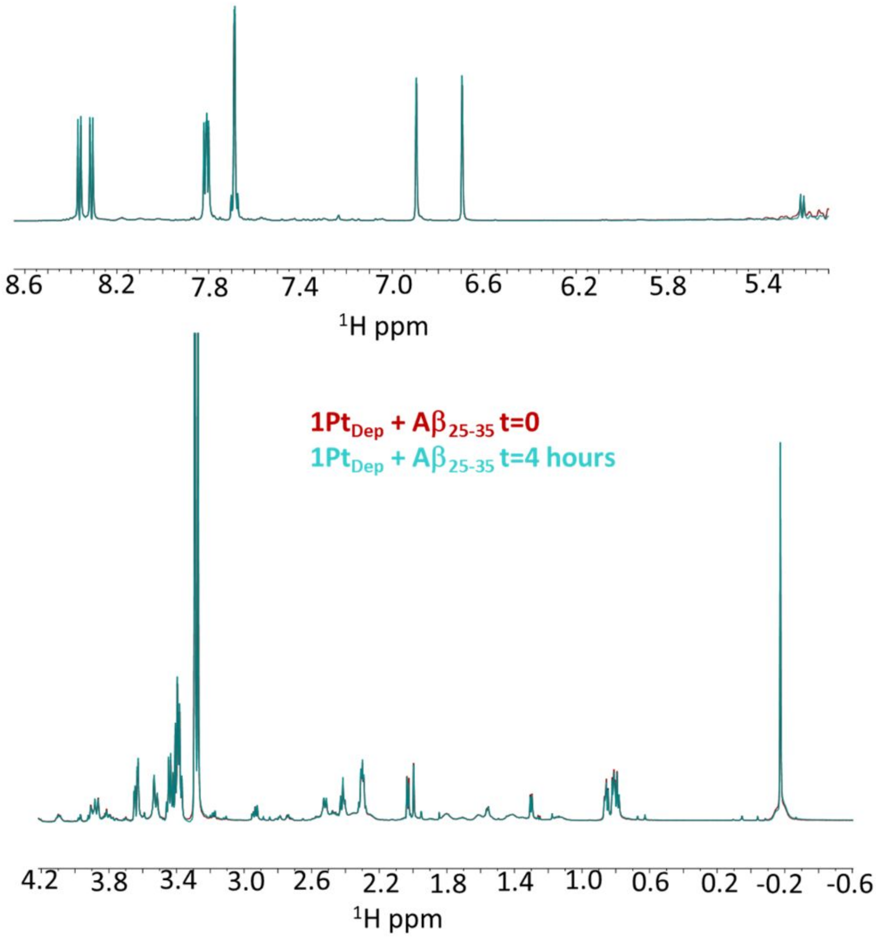

Figure S7. Overlay of 1D [^{1}H] NMR spectra of **1Pt$_{Dep}$** (2 mM) plus A$\beta_{25\text{-}35}$ (400 μM) at t=0 (red) and after four hours (cyan).

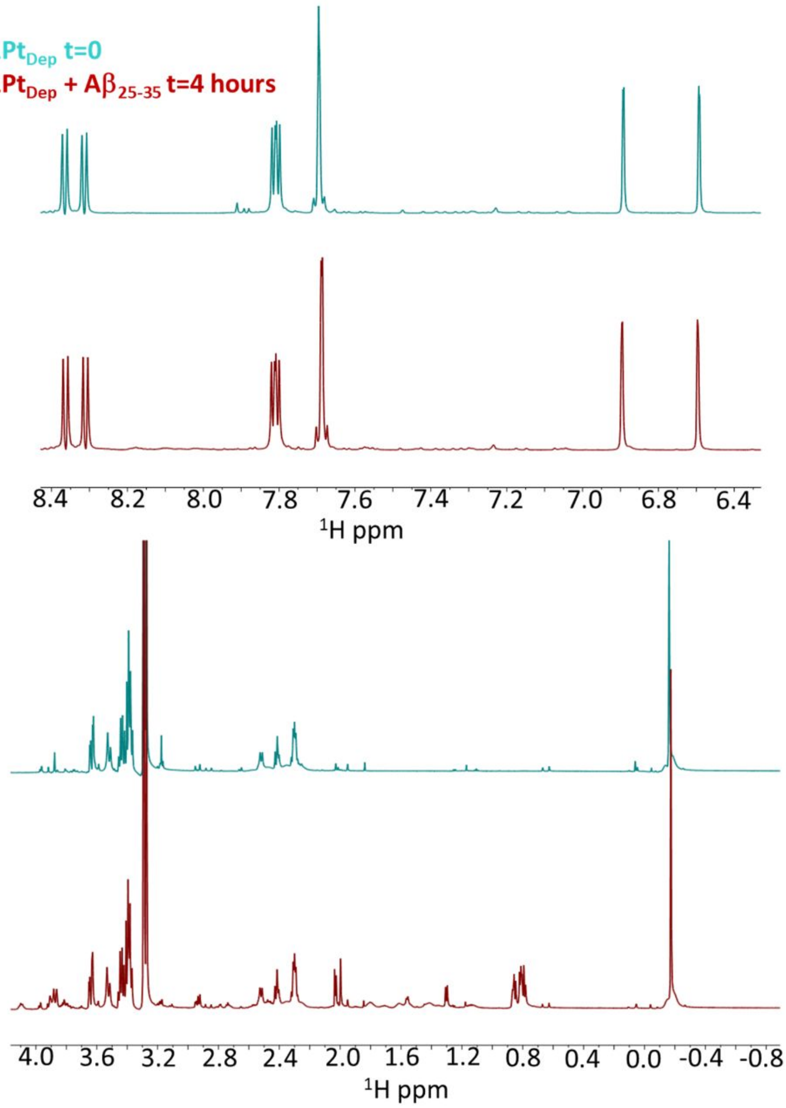

Figure S8. Comparison between 1D [^{1}H] NMR spectra of **$1Pt_{dep}$** alone at t=0 (cyan) and **$1Pt_{dep}$** in presence of $A\beta_{25\text{-}35}$ at t=4 hours (red).

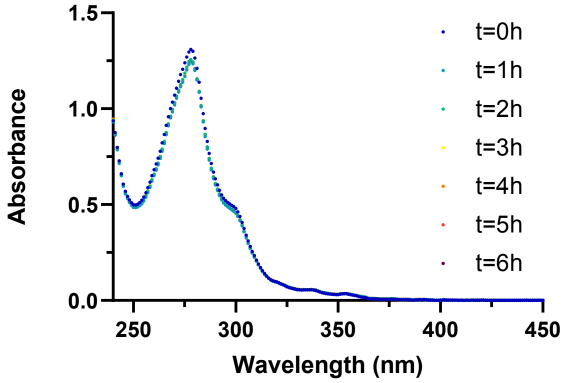

Figure S9. Time course UV-vis spectra of **1Pt** at 50 μM in 2% - DMSO phosphate buffer 10 mM at pH 7.4.

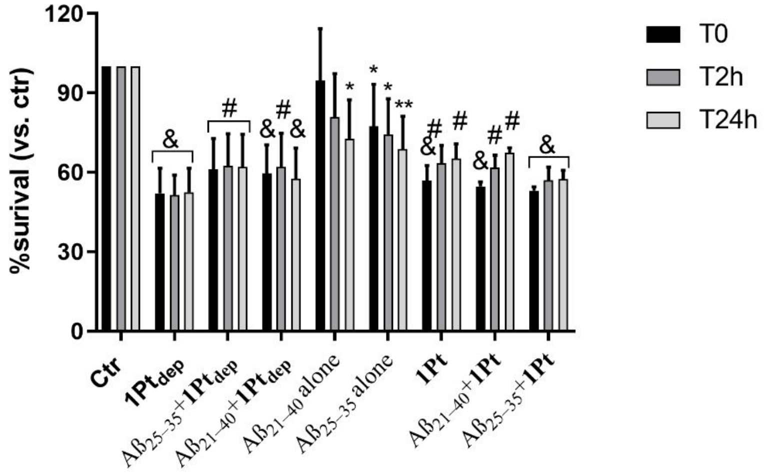

Figure S10. Cell survival of SH-SY5Y cells treated with $A\beta_{21\text{-}40}$ and $A\beta_{25\text{-}35}$ (100 μM) alone or with $\mathbf{1Pt_{dep}}$ or **1Pt** at 1:5 molar ratio at three time points: 0, 2 and 24 hours. Cells with peptides were incubated for 24h and then processed for MTT test. (*p< 0.05, **p<0.005, #p< 0.001, &p<0.0001 at statistical analysis of one experiment performed in triplicates).
